# Abnormal left ventricular global strain during exercise-test in young healthy smokers

**DOI:** 10.1038/s41598-020-62428-7

**Published:** 2020-03-30

**Authors:** Giuseppe Mandraffino, Egidio Imbalzano, Alberto Lo Gullo, Concetta Zito, Carmela Morace, Maria Cinquegrani, Francesca Savarino, Lilia Oreto, Clemente Giuffrida, Scipione Carerj, Giovanni Squadrito

**Affiliations:** 1 Department of Clinical and Experimental Medicine - Internal Medicine Unit - University of Messina, Messina, Italy; 2 IRCCS Centro Neurolesi Bonino Pulejo - Messina, Messina, Italy; 3 Department of Clinical and Experimental Medicine – Cardiology Unit - University of Messina, Messina, Italy; 4 Mediterranean Pediatric Cardiology Center, Bambino Gesù Pediatric Hospital, Taormina, Messina, Italy

**Keywords:** Biomarkers, Cardiology, Diseases, Health care, Medical research

## Abstract

Background. It is unknown how much precociously the cigarette smoking (CS) may compromise the integrity of the cardiovascular (CV) system. Myocardial function can be routinely assessed by conventional echocardiography, but abnormalities are only detected when somewhat a remodelling has already occurred. These limitations could be overcome by strain imaging. Methods. We evaluated whether young smokers with normal left ventricular (LV) geometry, wall motion and ejection fraction may present abnormalities in myocardial deformation, both at rest and during physical effort. We selected 50 young smokers with no additional CV risk factors, and 60 non-smokers to undergo a standardized exercise-test. Consistently, we evaluated the CV adaptation to exercise by both conventional echocardiography and speckle-tracking analysis (2D-STE). Results. We found no difference between smokers and controls regarding baseline characteristics; as expected, smokers presented with lower HDL-cholesterol (p < 0.005), and higher fibrinogen, C-reactive protein (CRP), and interleukin-6 (p < 0.001). Conventional echocardiography parameters were not different between groups, while we detected a different behaviour of global longitudinal strain (GLS), global circumferential strain (GCS) and twist by 2D-STE during exercise-test. Indeed, GLS, GCS and twist behaved differently during exercise test in smokers with respect to controls. We found an association between CS, inflammation and LV mechanics changes uncovered by physical effort, and regression analysis confirmed that the intensity of the exposure to cigarette smoking, together with the inflammatory status (CRP, fibrinogen and Il-6) plasma levels, drive this impairment. Conclusions. We confirm strain imaging (2D-STE) as a very useful tool to identify early changes in cardiac mechanics, as adaptation to exercise; our findings may reflect a very precocious functional abnormality in active smokers, likely long before structural damage occurs.

## Introduction

Cigarette smoking (CS) induces different adverse effects on cardiovascular (CV) system^1–3^, accordingly, in smokers, a cardiac involvement should be expected at any age^4,5^. Arterial stiffening and carotid artery wall thickening, together with increased inflammatory markers^4–6^, have been observed in very young smokers just a few years after starting smoking, suggesting early adverse effects of CS despite young age and short history of smoke exposure. Consistently, we have also shown in young people that smoke exposure increases the expression of monocyte biglycan (BGN), a central molecule in development of atherosclerosis^5^. Moreover, also young light smokers may present abnormal vascular response to exercise^7^. How precociously CS may impact on cardiac function, however, remains unknown. Conventional echocardiography has an important role in evaluating myocardial function, but it detects abnormalities only when a remodelling has already occurred. Strain imaging, by reflecting the intrinsic ability of the myocardium to deform during the cardiac cycle, could overcome these limitations ^8–10^. Complementary echocardiography software, like tissue Doppler Imaging (TDI) and particularly two-dimensional speckle tracking echocardiography (2D-STE) may identify early changes in left ventricular (LV) function by the measurement of global and regional longitudinal, radial and circumferential strain, and twist as well^8,9,11–16^. Global longitudinal strain (GLS) has been shown to identify abnormalities in cardiac function in different clinical settings, as arterial hypertension^14,17^, diabetes^18–20^, hypercholesterolemia^21^, liver^22^, myocardial steatosis^23^ and hypertrophic cardiomyopathies^24^. An altered GLS may predict long-term survival in patients with chronic ischemic cardiomyopathy^25^; moreover, GLS was estimated to have greater prognostic value than LV ejection fraction (LVEF) in predicting major adverse cardiac events^26^. Global circumferential strain (GCS) is also a predictor of altered systolic function in different conditions, including hypertension^14,17^, diabetes^27^ and hypercholesterolemia^21^ and may give information regarding the risk for heart failure and outcomes^28^. However, GCS seems to alter over time later than GLS and its reduction is also associated with a more extensive (transmural) myocardial damage^29^. Furthermore, LV torsional mechanics, including twist, appear to be impaired in a range of clinical conditions from those that cause minimal cardiac changes to advanced cardiac remodelling^30^. Therefore, overall GLS, GCS and twist, providing important insights into the pathophysiology of cardiac mechanics, are diffusely considered as sensitive markers of systolic function^31^. The aim of the present study was to evaluate by 2D-STE the adaptive response of LV deformation during bicycle exercise-test in young smokers with no additional CV risk factors and with normal LV systolic function, as assessed by conventional echocardiography. We also evaluated plasma levels of inflammatory molecules and the association with myocardial deformation during standardized exercise and with smoke exposure and with carotid arterial stiffness indices.

## Materials and methods

### Subjects

Fifty young healthy smokers aged between 18 and 36 years were enrolled in the study - median age 26.5 (10) years; males/females 24/26); exclusion criteria were: fasting glucose ≥105 mg/dl, plasma levels of cholesterol ≥230 mg/dl or low-density lipoprotein cholesterol (LDL-C) ≥160 mg/dl, triglycerides (TG) ≥250 mg/dl, systolic blood pressure (SBP) ≥140 mmHg and/or diastolic blood pressure (DBP) ≥90 mmHg, body mass index (BMI) ≥30, thyroid, liver or kidney diseases, abnormal electrocardiographic or echocardiography pattern, personal or familiar history of CVD, or personal history of alcohol consumption; women taking hormone-based therapy were not included in the study. Sixty non-smoking subjects matched for age and gender distribution were enrolled as control subjects. Only active smokers were included in the study; as control subjects, only never smoked were included. Former smokers were not included in the study. Moreover, in order to avoid any acute effect of cigarette smoking, included subject were recommended not to smoke the day of the clinical evaluation, from the midnight of the day before. All the patients were Caucasian, living in the surrounding of Messina (Sicily) or Reggio Calabria (Calabria), Italy, referred to our outpatient clinic requesting an evaluation for physical fitness assessment (non-competitive activity). All analyses were performed on a venous blood sample taken at the medical centre. Total cholesterol (TC), TG, high-density-lipoprotein-cholesterol (HDL-C), glucose and fibrinogen were measured by routine methods. LDL-C was calculated using the Friedewald formula. High-sensitivity C-reactive protein (HsCRP) was determined using an immunoturbidimetric latex assay kit.

Written informed consent was obtained from all subjects, and the study conforms to the ethical guidelines of the 1975 Declaration of Helsinki; the study protocol was approved by local Ethics Committee (Intercompany Ethical Committee of Messina, protocol number 85/15).

### Smoke exposure index

Smoke exposure index (SEIx) was estimated as already reported elsewhere^32^. Briefly, each smoker had a score between 0 and 1 (0 = no exposure;1 = maximum exposure), accounting for number of smoked cigarettes per day and duration of smoke habit by applying the following formula: (n × 30 × m)/h, where *n* is the number of cigarettes per day, 30 as average days per month, *m* represents the duration of CS expressed in months and *h* is the highest value obtained from (n × 30 × m). Second-hand smoke exposure was not taken into account.

### Ultrasound study

Carotid ultrasonography evaluation for cIMT and AS assessment has already been described^33^. Briefly, semi-automated cIMT evaluation was performed by using Aloka ProSound ALPHA10 (Hitachi Aloka Medical, Ltd, 6-22-1, Mure; Mitakashi, Tokyo, Japan) with a 7–15 MHz linear array transducer; according to ESC/ESH guidelines, we considered a mean cIMT ≥0.9 mm or plaque as carotid wall thickening. Augmentation Index (AIx) and Pulse Wave Velocity (PWV) as AS indices were measured automatically by the “eTRACKING” software implemented in the ultrasound machine. The instrumental evaluations were repeated twice in duplicate by two experienced independent operators. The interobserver variability of IMT measurements was 0.018 mm; the intraobserver variability was 0.011 mm. About AIx and PWV, the interobserver variability was 9.7% and 7.3%, respectively; the intraobserver variability was 8.4% and 6.4%, respectively.

Echocardiography examination was performed using a VIVID-7 ultrasound machine (GE Vingmed Ultrasound, Horten, Norway) equipped with a phased-array transducer, and stored on a dedicated workstation (EchoPAC, version 8.0.0; GE Medical Systems, Horten, Norway) for off-line analysis. All measurements were performed according to the recommendations of the American Society of Echocardiography on three averaged cardiac cycles^34^. In accordance, LV mass was determined with the area–length method, and the mass index (LVMI) was calculated as the LV mass/body surface area (g/m^2^) ratio; LV hypertrophy was diagnosed as LVMI >102 g/m2 in men and >88 g/m^2^ in women; a cut-off value of ≥0.45 for the relative wall thickness (RWT) was considered to define a concentric remodelling; mitral flow peak velocities (E and A) and E/A ratio were measured using pulsed wave Doppler. Furthermore, from stored colour tissue Doppler imaging loops, the value of E’ was obtained by averaging the peak early-diastolic velocities calculated at the level of the septal, lateral, anterior, and inferior corner of the mitral annulus. The E/E’ ratio was also included as an estimate of LV filling pressure. To complete the analysis of LV systolic function, myocardial deformation was assessed by STE and automated function imaging for the evaluation of global and regional longitudinal (GLS) and circumferential (GCS) strain, as previously described^14^. Automated function imaging was performed on apical long-axis, 4-chamber, and 2-chamber views, following an on-screen guided work flow. The results were presented as a bull’s-eye display showing color-coded and numeric values for peak systolic GLS and GCS. A detailed description on how all these measurements were made was previously shown^14,15^. Each subject underwent exercise-test, by using upright cycloergometer, according to an incremental step protocol with an increase of 25 Watt every three minutes^35^. Criteria for test interruption were those conventionally recommended^35^. Images and videoclips of parasternal long and short axis view, at basal, mid and apical levels, and of apical four-, two-, and three-chambers view were acquired at rest, 50 watt, 100 watt, and recovery. A frame rate >70 fps was employed. Strain analysis was performed offline by using Echopac. Endocardial border was manually traced from apical views, automatically obtaining the calculation of a region of interest comprised between endocardial and epicardial layers. Tracking quality was verified for each segment and low quality images were excluded. Global values of longitudinal strain from each apical view were calculated through an Automated Function Imaging analysis. LV twist was defined by the difference (in degrees) between apical rotation and basal rotation at isochronal time points. Two independent observers, unaware of the clinical conditions of patients, evaluated the recordings and calculated the parameters.

### Statistical methods

The Kolmogorov-Smirnov test verified that several variables had a non-normal distribution; consequently, also given the relatively small size of our sample, we chose a non-parametric statistical approach. Accordingly, data were expressed as median (IQR); comparisons between controls and patients were carried out by the Mann-Whitney test, and categorical variables were tested by Chi square; the risk depending on the variable “smoke” has also been estimated by Odds Ratio. The correlations among the variables were assessed by Spearman’s test. We also estimated multiple regression models to assess the contribution of the main study variable on the chosen response variables (strain analysis related variables). In details, we estimated potential predictor among the independent variables we considered to have a biological plausibility in affecting LV performance: Hs-CRP; IL6; LDL-C; HDL-C; SBP50w; DBP50w; HR50w; Fibrinogen; SEIx. GLS, GCS and twist (at second step) were tested as dependent variable, individually, one for each estimated model. A two-tailed alpha of 0.05 was used to denote statistical significance.

To perform the statistical analyses, we used the SPSS statistical package, version 17.0 (Chicago, IL, USA).

## Results

### Baseline evaluation

Table 1 shows baseline characteristics of smokers and control subjects; no difference was detected as regards BMI, BSA, SBP and DBP values, TC, TG, LDL-C and fasting glucose levels; in smokers plasma HDL-C was lower (p = 0.005), while inflammatory markers were significantly higher (HsCRP: p < 0.005; fibrinogen: p < 0.035; Il-6: p < 0.001). No differences were detected between groups as regards conventional cardiac and vascular ultrasound parameters; in addition, AIx and PWV were also estimated at baseline in smokers and controls: −3.5(11) vs −6.1 (6) and 6.7(1.6) vs 5.4 (1); p = 0.008 and p < 0.001, respectively (Table 2). In Table 3 we report the results from strain analysis during the exercise test; similar values of basal GLS, GCS and twist were detected in smokers and controls: −19.5(2.5)% vs −19.7(2.2)%, p = ns; −20.7(2.7)% vs −21.1(2)%, p = ns; 12.4(3.5)% vs 11.7(4.4)%, p = ns. Intra- and inter- observer variability was assessed through intra-class correlation coefficients (ICC); were ≥0.84 for all global strain measurements, with average coefficients of variation (CV) of ≤4% for global longitudinal and circumferential strain. The average CVs were <6% for global longitudinal and circumferential strain. Inter- and intra-observer reproducibility findings were similar in analyses adjusting for frame rate.

**Table 1:** Characteristics of study population (smokers and controls).

	Controls	Smokers	p
Number	60	50	
Gender (m/f)	33/27	24/26	
	**Median (IQR)**	**Median (IQR)**	**Mann-Whitney**
Age (years)\	26.5 (12)	26.5 (16)	0.415
Age range (yrs)	18–36	18–34	—
BMI (m^2^)	24.3 (6)	25.6 (3.4)	0.283
BSA (m^2^)	1.88 (0.3)	1.9 (0.3)	0.189
SBP (mmHg)	120 (20)	122 (30)	0.404
DBP (mmHg)	70 (15)	70 (21)	0.185
TC (mg/dl)	160 (49)	160 (26)	0.943
HDL-C (mg/dl)	43 (8)	41 (9)	**0.005**
TG (mg/dl)	135 (33)	140 (33)	0.065
LDL-C (mg/dl)	90 (49)	90 (28)	0.822
Glucose (mg/dl)	81 (11)	85 (15)	0.087
Hs-CRP (mg/dl)	0.6 (0.3)	0.8 (0.4)	**0.002**
fibrinogen (mg/dl)	241 (70)	260 (109)	**0.035**
IL-6 (ng/dl)	0.85 (0.7)	1.2 (0.3)	**0.001**
Cigarettes per day	—	20 (10)	—
Smoking initiation (age in years)	—	19 (4)	—
Smoking duration (months)	—	84 (84)	—
SEIx	—	0.25 (0.30)	—

Values are Median (IQR). BMI: body mass index; SBP: systolic blood pressure; DBP: diastolic blood pressure; BSA: body surface area; TC: total cholesterol; HDL-C: high-density lipoprotein-cholesterol; TG: triglycerides; LDL-C: low-density lipoprotein-cholesterol; HsCRP: high-sensitivity C-reactive protein; Il-6: interleukin-6; SEIx: smoke exposure index. p: significance level Mann-Whitney test, Smokers vs Controls.

**Table 2:** Conventional echocardiography parameters.

	Controls	Smokers	p
Number	60	50	ns
Gender (m/f)	33/27	24/26	ns
	**Median (IQR)**	**Median (IQR)**	**Mann-Whitney**
LV-EDV (ml)	84 (27)	84 (26)	0.518
LV-ESV (ml)	28 (9.5)	32 (9)	0.297
EF (%)	63.9 (6.9)	62.5 (5.9)	0.425
IVS (mm)	9 (2.5)	9 (3)	0.403
LVMIx (g/m^2^)	71.8 (26.9)	70.6 (16.5)	0.728
RWT	0.36 (0.2)	0.35 (0.1)	0.743
E (cm/sec)	90 (15)	90 (29)	0.831
A (cm/sec)	52 (23.5)	52 (16)	0.143
E/A	1.7 (0.7)	1.6 (0.5)	0.220
S’(cm/sec)	7 (1.5)	7 (3.5)	0.788
E’ (cm/sec)	10.25 (2)	11.2 (3.1)	0.246
E/E’	7.5 (3)	8.4 (3.1)	0.646
cIMT	0.8 (0.28)	0.82 (0.3)	0.29
AIx	−6.17 (6)	−3.44 (11)	0.008
PWV	5.4 (1)	6.71 (1.6)	<0.001

Values are the Median (IQR). LV-EDV: left ventricle end diastolic volume; LV-ESV: left ventricle end systolic volume; LV-SV: left ventricle stroke volume; EF: ejection fraction; IVS: interventricular septum; E: peak early mitral flow velocity; A: peak late mitral flow velocity; E’: peak early diastolic mitral annular velocity; S’: peak systolic mitral annular velocity; p: significance level for Mann-Whitney test, Smokers vs Controls.

**Table 3:** LV Strain values, blood pressure and heart rate during exercise test.

	GLS					GCS					Twist				
	C	(IQR)	S	(IQR)	p	C	(IQR)	S	(IQR)	p	C	(IQR)	S	(IQR)	p
Basal	−19.7	(2.2)	−19.5	(2.5)	0.23	−21.1	(2)	−20.7	(2.7)	0.66	11.7	(4.4)	12.4	(3.5)	0.13
50 W	−21.9	(1.3)	−19.7	(2.0)	**<0.001**	−22.3	(2)	−21.6	(2)	**<0.001**	13.3	(4.3)	14.9	(4.7)	**0.03**
100 W	−21.3	(2.4)	−20.8	(2.7)	**0.045**	−22.5	(2.2)	−22.1	(2.7)	0.13	14.6	(4.5)	16.1	(4.5)	**0.008**
Recovery	−19.4	(2.9)	−19.4	(2.3)	0.8	−21.6	(2.1)	−21.3	(2.3)	0.02	11.8	(4)	15.8	(4.5)	**<0.001**
	**SBP**					**DBP**					**HR**				
	**C**	**(IQR)**	**S**	**(IQR)**	**p**	**C**	**(IQR)**	**S**	**(IQR)**	**p**	**C**	**(IQR)**	**S**	**(IQR)**	**p**
Basal	119.5	(17)	123.5	(26)	0.27	69	(12)	69	(21)	0.5	79	(17)	79.5	(18)	0.9
50 W	163	(20)	173.5	(30)	**0.01**	84	(15)	91	(21)	**<0.001**	138	(10)	149.5	(8)	**<0.001**
100 W	174	(19)	183.5	(27)	**0.01**	95	(13)	99	(18)	**<0.001**	166	(12)	172.4	(11)	**<0.001**
Recovery	116	(21)	124.5	(29)	**0.01**	70	(13)	73	(20)	**<0.001**	85	(15)	88.5	(18)	**0.14**

Study parameters during 4-step exercise test: Values are median (IQR). GLS: Global longitudinal strain; GCS: Global circumferential strain. SBP: systolic blood pressure. DBP: diastolic blood pressure. HR: heart rate. p: significance level for Mann-.Whitney test; Smokers vs Controls.

### Exercise-test evaluation

All subjects, smokers and controls ended the exercise by completing the planned steps of 100 W. No change on ECG and LV wall motion scores were detected during the effort, and a normal behaviour of the BP and heart rate (HR) was recorded for the whole duration of the test. Indeed, median systolic BP at peak exercise was 183.5(30) mmHg in smokers and 174(20) mmHg in controls (p = 0.01) and mean diastolic BP was 99(21) mmHg in smokers and 95(15) in controls (p = 0.01). HR at the peak of exercise was 172.4(8) bpm and 166(10) bpm in smokers and in controls respectively (p < 0.001). There was no need to interrupt the exercise for symptoms and/or arrhythmias in any case.

**Figure 1:**
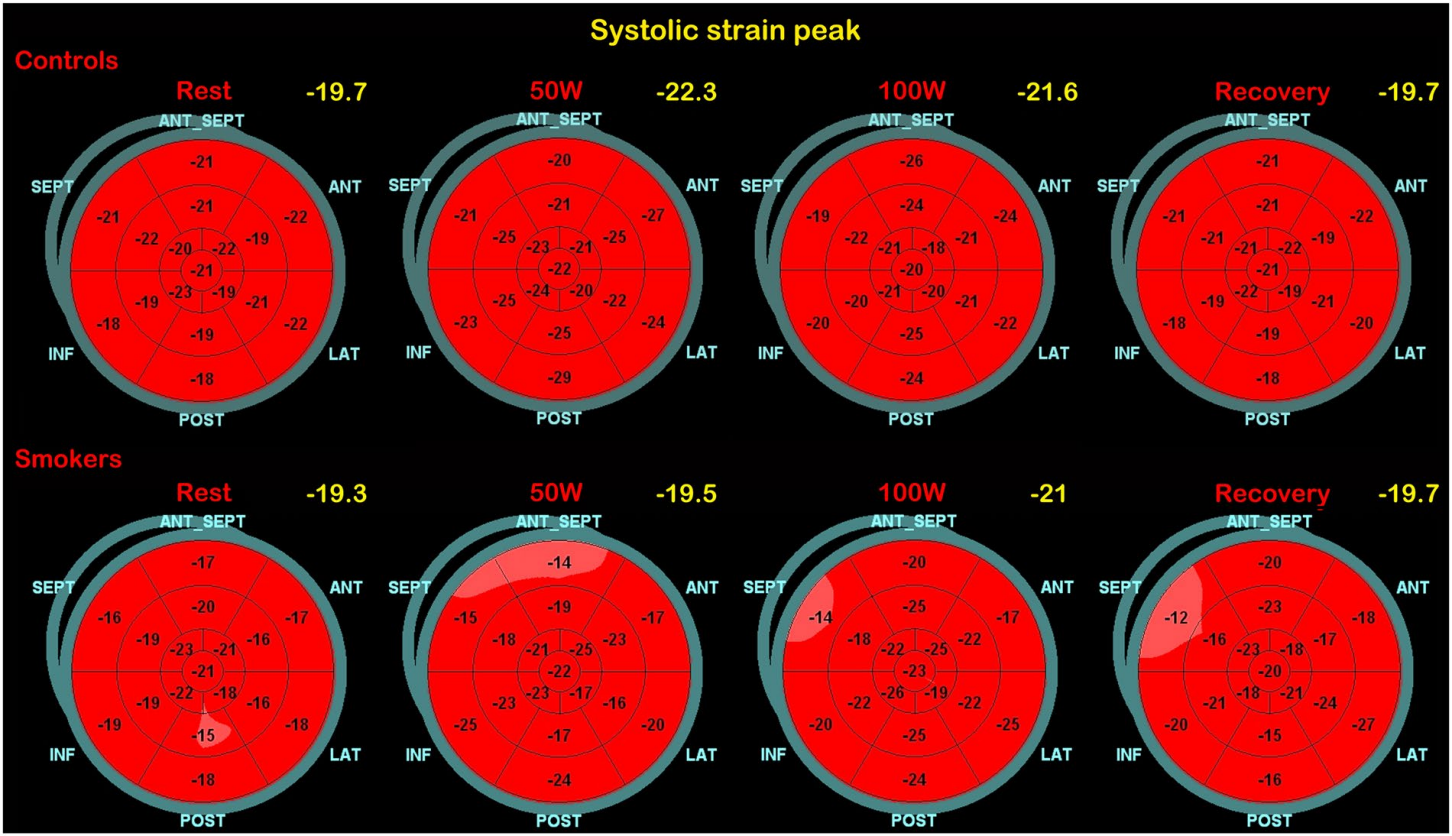
The bull’s eyes represent the regional systolic strain peak by time (rest, 50 W, 100 W, and rest); upper panel: controls (representative exam); lower panel: smokers (representative exam); in yellow the mean values step by step are also reported.

**Figure 2:**
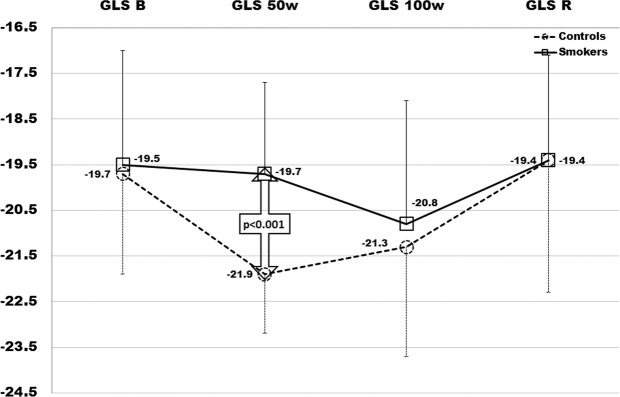
GLS of Smokers (squares and continue line) and Controls (rounds an dashed line) at each time-point (baseline, 50 W, 100 W, recovery). The statistical significance is reported for a two-sided alpha level of 0.05.

**Figure 3:**
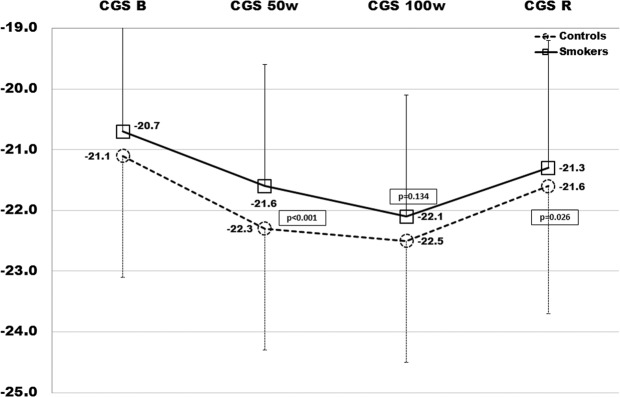
GCS of Smokers (squares and continue line) and Controls (rounds an dashed line) at each time-point (baseline, 50 W, 100 W, recovery). The statistical significance is reported for a two-sided alpha level of 0.05.

**Figure 4:**
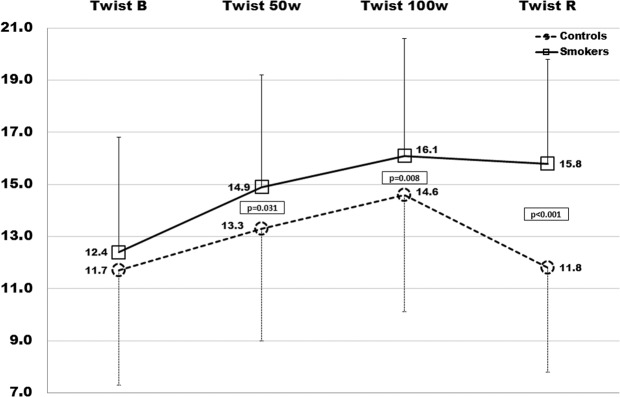
Twist of Smokers (squares and continue line) and Controls (rounds an dashed line) at each time-point (baseline, 50 W, 100 W, recovery). The statistical significance is reported for a two-sided alpha level of 0.05.

Regarding systolic function, we observed that EF remained within the normal range compared to the baseline both in smokers and controls with a mean value at 100 W of 69(2)% and 68.4(1.7)%, in smokers and controls respectively (p = ns). However, by analysing the LV systolic mechanics changes during exercise through STE, we observed a different behaviour mainly of GLS, but also of GCS and twist, in smokers with respect to non-smokers. In Fig. 1 the regional systolic strain peak by time (rest, 50 W, 100 W, and recovery) is compared between smokers and controls (represented by bull’s eye). Of note, since longitudinal strain represents the systolic shortening of myocardial fibers, it is conventionally expressed as a negative value: a more negative value expresses a better performance. Controls showed a quick and significant increase of GLS values at 50 W: −21.9(1.3)%, p < 0.001, and then a slow trend back to the baseline values 100 W: −21.3(2.4)%; recovery: −19.4(2.9)%), while smokers showed a delayed increase of strain values from 1^st^ to 2^nd^ step of exercise-test, re-aligning at 3^rd^ and 4^th^ step to the control values (Fig. 2). Although similar at baseline, GCS values in smokers changed less markedly during exercise (Fig. 3**)**, whereas twist values increased much more by time-points, including at recovery, (Fig. 4) with respect to controls. Table 3 reports the median values of LV strain parameters together with BP and HR point by point (basal, 50 W, 100 W and recovery).

At baseline, HsCRP, Il-6, and fibrinogen were all correlated among them; also, inflammatory markers were correlated with the number of cigarettes smoked per day, the smoking duration, and SEIx; basal values of GLS, GCS and twist were not significantly correlated with other study variables.

Moreover, interdependence analysis showed that GLS Δ% change from 1^st^ to 2^nd^ step of exercise-test (Δ50w) was inversely correlated with inflammatory markers and smoking parameters, and positively with HDL-C values (Table 4). Lower longitudinal strain values were correlated to higher inflammatory markers, and also with SEIx; moreover; GCS showed similar relationships. We found no significant correlation between LV strain parameters (both baseline and during exercise) and systolic or diastolic blood pressure values or heart rate (both baseline and during exercise).

**Table 4:** Relationships among the variables (smokers; n = 50).

	Fibrinogen	Hs-CRP	Il-6	HDL-C	SEIx	Smoking duration	Cigarettes per day
Cigarettes per day	**rs 0.334**	**rs 0.337**	rs 0.165	rs −0.066	rs 0.463	rs 0.197	—
	**p** = **0.018**	**p** = **0.017**	p = 0.252	p = 0.650	p < 0.001	p = 0.170	—
Smoking duration	**rs 0.399**	**rs 0.383**	rs 0.209	**rs −0.355**	rs 0.950	—	rs 0.197
	**p** = **0.004**	**p** = **0.006**	p = 0.145	**p** = **0.011**	p < 0.001	—	p = 0.170
SEIx	**rs 0.457**	**rs 0.439**	rs 0.233	**rs −0.388**	—	rs 0.950	rs 0.463
	**p** < **0.001**	**p** < **0.001**	p = 0.103	**p** < **0.005**	—	p < 0.001	p < 0.001
GLS Δ50w	**rs −0.413**	**rs −0.428**	**rs −0.499**	**rs 0.380**	**rs −0.353**	**rs −0.349**	rs −0.120
	**p** = **0.003**	**p** = **0.002**	**p** < **0.001**	**p** < **0.005**	**p** = **0.012**	**p** = **0.015**	p = 0.408
GCS Δ50w	**rs −0.391**	**rs −0.434**	**rs −0.512**	**rs 0.396**	**rs −0.353**	**rs −0.352**	rs −0.178
	**p** = **0.004**	**p** = **0.002**	**p** < **0.001**	**p** < **0.005**	**p** = **0.012**	**p** = **0.012**	p = 0.355

HsCRP: high-sensitivity C-reactive protein; Il-6: interleukin-6; HDL-C: high-density lipoprotein-cholesterol; SEIx: smoke exposure index. GLS/GCS Δ50w: change (%) between 1st and 2nd step during exercise test. p: significance level for Spearman’s test (rs: correlation coefficient).

We estimated two multiple regression models to assess the contribution of Hs-CRP, fibrinogen, Il-6, LDL-C, HDL-C and SEIx to the main findings of our study: the abnormal value of GLS and GCS at the 1^st^ step of the exercise protocol (50w). We found a significant dependence of both GLS and GCS from SEIx (Table 5). No predictors were found for Twist (50w) among the putative variables entered in the regression model. We also found that Hs-CRP levels are significantly associated to SEIx (beta: 0.494, p < 0.001).

**Table 5:** Multiple regression analysis.

Dependent variable	Predictors	B	95% CI for B	Beta	T	p
GLS Δ 50w	SEIx	5.368	2.993; 7.744	0.548	4.544	<0.001
	Hs-CRP	2.711	0.638; 4.783	0.345	2.631	0.011
GCS Δ 50w	SEIx	3.078	1.333; 4.822	0.465	3.547	=0.001
Hs-CRP	SEIx	0.614	0.300; 0.928	0.494	3.932	<0.001

Multiple regression analysis for GLS Δ50w. B, unstandardized regression coefficient; 95%CI, 95% Confidence Interval for B; Beta, standardized regression coefficient; T, t-test for Beta; p, p-value for significance. Independent variables tested: Hs-CRP; IL6; LDL-C; HDL-C; SBP50w; DBP50w; HR50w; Fibrinogen; SEIx.

## Discussion

In the present study we show that young healthy smokers with no additional risk factors for atherosclerosis present a pattern of altered LV strain during physical exercise, in addition to having increased markers and mediators of inflammation, including increased CRP, fibrinogen and IL-6 plasma levels, and altered arterial stiffness indices at baseline. Although strain values during exercise were substantially within a range reported as “normal”, following the current literature^36–38^, at each time-point in both groups, smokers presented lower (worse) values of GLS, GCS and twist with respect to controls. In particular, despite the time-course curves of GCS showed a behaviour similar to controls, even if on the edge of the normal range, the curve of GLS showed a trend markedly different, as to suggest a delayed adaptation of the heart to acute hemodynamic overload. As regards LV twist behaviour, we also noted that in smokers the mean values remained significantly different from controls especially during the recovery step (maybe suggesting a compensatory mechanism acting to preserve systolic output despite the impairment of GLS).

Furthermore, we found different blood pressure behaviour in smokers as compared to controls, likely due to endothelial dysfunction in the smoker group; the slight but significant increase of PWV, in fact, although within the normal range^39^, could be the expression of early endothelial dysfunction in smokers group.

Current smokers have been already reported to have an inflammatory profile; it is also known that active CS stimulates transcription factors, pro-inflammatory gene and receptors, promoting the release of inflammatory molecules and acute phase proteins and inducing functional and structural damage in arteries^1–3,40,41^ and heart^42,43^. The association between inflammation and subclinical atherosclerosis, including flow-mediated dilation and arterial stiffening and coronary artery calcium^44^ has been also reported in smokers. In addition, we found that high CRP, fibrinogen and Il-6 levels and especially smoke-related parameters are correlated with higher risk of a worse change from 1^st^ to 2^nd^ step of exercise-test (Δ50w) of GLS. Also, we confirmed that Hs-CRP plasma levels, but also fibrinogen and Il-6 plasma levels, are associated with the smoke-related parameters (mainly SEIx). We believe that this further finding strengthens the link among CS, inflammation and cardiac performance.

The underlying mechanisms of many CS effects on the heart and blood vessels remain to be clarified; however, several studies have shown that CS, and also nicotine directly, may induce cardiac toxic effects^42,45–49^; smoking indeed has been found to be a risk factor for cardiac hypertrophy and dysfunction, independently of other risk factors as hypertension and atherosclerosis^49^. Thus, a precocious cardiac involvement should be expected even in young smokers ^7,12^. Actually, myocardial mechanics were recently investigated by 2D-STE in young heavy smokers^12^ at baseline and after acute smoking: although systolic and diastolic function did not differ between smokers and controls, neither after acute smoke exposure, as assessed by conventional echocardiography, 2D-STE revealed an impaired diastolic relaxation as possible early sign of cardiac dysfunction, with no significant difference as regards basal GLS; moreover, diastolic relaxation appeared to worsen by acute smoking^12^. Here, we report in young healthy smokers a cardiac dysfunction, as estimated by 2D-STE, with no overt change in strain values at baseline; to the best of our knowledge, this is the first study showing in young smokers a precocious and subclinical altered systolic mechanics, uncovered by physical effort.

Different mechanisms may be potentially involved in determining early, subtle and subclinical changes in cardiac function in our smokers, including reduced NO availability ^2,45,46^ and impairment of NO- mediated pathways modulating CV homeostasis and myocardial adaptation to physical effort^50^, disturbed myocardial energy metabolism^42,49^, autonomic activation^51^, subtle changes in coronary blood flow^52,53^ and increased LV stiffness leading to elevated filling pressures and delayed LV intrinsic adaptation to hemodynamic demand^7,12^. Moreover, we found that smoking status was correlated with altered strain and twist course and that a greater smoke exposure, together with higher fibrinogen and lower HDL-C levels, predict the delayed increase of longitudinal strain during exercise. So, it seems likely that smoke-induced changes, including inflammation and oxidation, could play a role in myocardial systolic dysfunction. Of course, considering the role of afterload on myocardial deformation, also the increased arterial stiffness and the different blood pressure behaviour we found in our young smokers could play a role in affecting left ventricular strain properties.

This study however presents several limitations. The first limitation is the small sample size, due to strict exclusion criteria: we selected 110 healthy young subjects, 50 smokers were compared to 60 non-smokers controls; however, absolute values, and also the shape of the time-points curve and GLS, GCS and twist values, although in the normal range, appear to be statistical different. Also, smoking duration shows a wide range; due to the relatively small sample size, any correction for this variable or a stratification according to its tertiles could be statistically poor; however, these limitations may be overcome by larger study population. Anyway, our observation, while considering everything, seems to suggest that more than smoking duration, the subclinical mechanical systolic abnormalities may be due to the intensity of cigarette smoking (as expressed by the SEIx), and the related inflammatory status should be also considered to understand the changes of ventricular performance during exercise.

## Conclusions

Our findings indicate that young smokers may show early signs of a maladaptive response of the heart to physical stress, before changes can be seen at rest. However, we are not able to show the cause and mechanisms of altered LV systolic deformation, nor we know if these abnormalities may reflect subclinical structural heart damage or are just the result of change in load during exercise when arterial distensibility appears impaired, although BP values remain within the normal range. In smokers 2D-STE detects early changes in cardiac systolic mechanics much earlier than traditional echocardiography, and long before these changes become clinically apparent; however, we can suggest that the pro-inflammatory status associated to CS (mainly due to the intensity of smoke exposure) may be involved in this abnormality. Stop smoking as soon as possible, even at a young age, before permanent damage can occur, remains anyhow a major recommendation. Future studies should test whether smoking cessation is able to restore the weakened LV systolic adaptation evoked by hemodynamic overload and altered vascular response during physical stress in smokers.
